# Nanomedicine Penetration to Tumor: Challenges, and Advanced Strategies to Tackle This Issue

**DOI:** 10.3390/cancers14122904

**Published:** 2022-06-13

**Authors:** Muhammad Usman Munir

**Affiliations:** Department of Pharmaceutical Chemistry, College of Pharmacy, Jouf University, Sakaka 72388, Aljouf, Saudi Arabia; mumunir@ju.edu.sa

**Keywords:** nanomedicine, drug delivery, tumor microenvironment, drug penetration, polymeric nanoparticle, cancer therapy, nanocarriers, siRNA, anticancer, extra-cellular matrix

## Abstract

**Simple Summary:**

Scientists have been working on the development of nanomedicine-based tumor therapy, rather than conventional treatment, to treat cancer for several years. Unfortunately, biological barriers hinder the delivery of nanomedicine to tumors. There are different strategies applied by researchers to improve the nanomedicine penetration to tumors, such as the modification of nanoparticle features and controlling the cancer micro-environment. However, complicated cancer micro-environments and delivery problems prevent these approaches from achieving optimal results. Changes in the extra-cellular matrix or blood vessels modify tumor microenvironments in a way that provides improved nanomedicine penetration and distribution but not to an optimal level. Herein, we reviewed the challenges of nanomedicine penetration to tumors along with possible approaches to deal with this concern.

**Abstract:**

Nanomedicine has been under investigation for several years to improve the efficiency of chemotherapeutics, having minimal pharmacological effects clinically. Ineffective tumor penetration is mediated by tumor environments, including limited vascular system, rising cancer cells, higher interstitial pressure, and extra-cellular matrix, among other things. Thus far, numerous methods to increase nanomedicine access to tumors have been described, including the manipulation of tumor micro-environments and the improvement of nanomedicine characteristics; however, such outdated approaches still have shortcomings. Multi-functional convertible nanocarriers have recently been developed as an innovative nanomedicine generation with excellent tumor infiltration abilities, such as tumor-penetrating peptide-mediated transcellular transport. The developments and limitations of nanomedicines, as well as expectations for better outcomes of tumor penetration, are discussed in this review.

## 1. Introduction

Cancer is a disease involving the manifestation of uncontrolled cell division, resulting in the suppression or proliferation of tumor cells [1]. Nanoparticle (NP)-based tumor therapy has been developing for many years, and it has made significant progress in transporting therapeutic substances, such as peptides, chemotherapeutic agents, genes, proteins, and molecular targeting agents [2,3,4]. Some biological barriers are present that halt the delivery of nanomedicines; however, there are ongoing attempts to overcome these hurdles. Various nano formulated drugs are certified by the US FDA, such as irinotecan liposome (Onivyde^®^), liposome of doxorubicin (DOXIL^®^), and albumin-bound paclitaxel (Abraxane^®^), which have prolonged circulation and less toxicity. Furthermore, nanomedicines increase the tumor aggregation of a drug by passive or active targeting [5,6,7]. However, these nanomedicines do not produce extraordinary clinical results; a few of them were even ineffective in clinical trials, such as BIND-014 [7]. Low tumor penetration is one of the leading dilemmas of nanomedicines.

The nanomedicine delivery at the tumor site is inadequate and associated with the features of NPs and tumor microenvironments. Firstly, the diverse blood supply that is adequate at the margins of tumors and reduced in the center demands that the NP travels a more considerable distance to the tumor center [8]. Subsequently, interstitial fluid pressure (IFP) raises from the tumor’s margins to its center, restraining NP diffusion to thick tumor areas after the eruption from peripheral vessels [9]. Furthermore, the deep extra-cellular matrix (ECM) hinders the NP passage by tiny holes inside the matrix [10,11,12]. In addition to biological barriers, the distinctive characteristics of NPs compared to tiny molecules result in considerable challenges. For instance, nanosized delivery systems are less susceptible to extravasation and diffusion after their aggregation at the tumor margin. Furthermore, complex and conflicting relations exist between NP features and in-vivo tumor penetration at various stages of the drug transport method. Generally, inadequate nanomedicine delivery at a tumor site is associated with the intrinsic features of NPs as well as tumor microenvironments.

**Figure 1:**
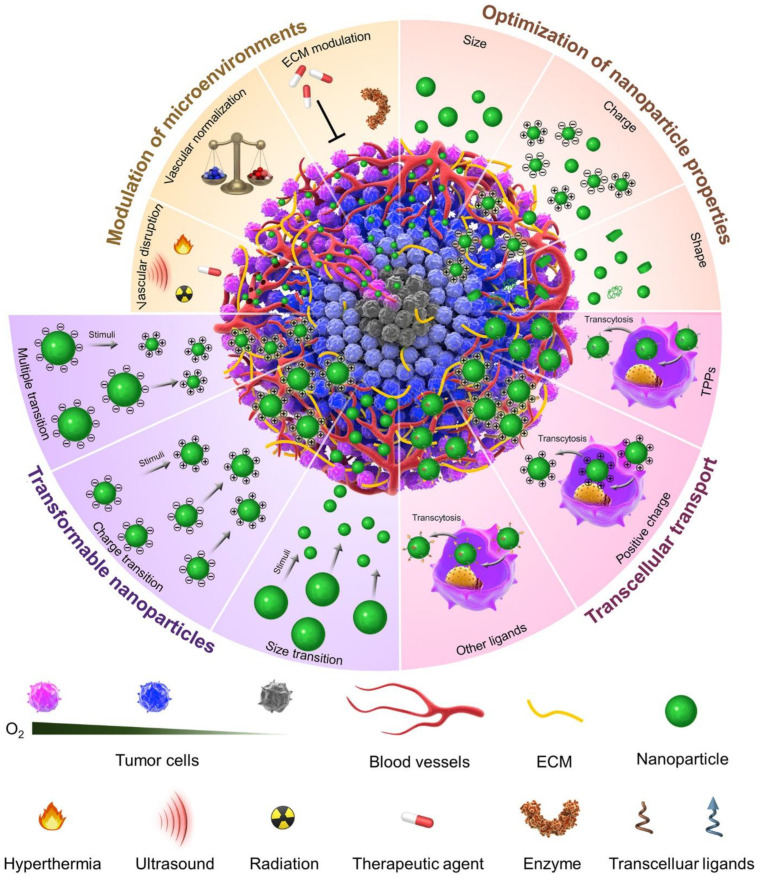
Attempts to improve the nanomedicine penetration, primarily by altering the nanoparticle’s features and modulating tumor microenvironments. Re-printed with permission from reference [13]. Copyright (2019), Elsevier.

Numerous attempts have been made to enhance nanomedicine penetration, mainly by modifying the features of nanoparticles and modulating the tumor microenvironments, as shown in Figure 1 [13,14,15,16,17,18]. However, these strategies have been restricted by the delivery cascades and complex tumor microenvironments. However, modulation of tumor ECM or blood vessels changes the tumor microenvironment and permits better diffusion and distribution of nanoparticles. Still, the alteration of the external force might disrupt the microenvironment, eventually restraining recurrent treatment or inducing tumor metastasis. In contrast, nanoparticles’ definite shape, charge, and size can be optimized by screening to increase penetration tumor site. Still, the optimized features depend upon the kind of tumor and should be examined further as they can obstruct other drug delivery methods. Several tailored nanoplatforms with penetration-aided ligands or transformable features, such as reversible charge or shrinkable size, have been inspected to cope with these restrictions. This article is concerned with improving NP delivery at the tumor sites with a focus on optimally designed nanoplatforms.

## 2. Tumor Penetration Problems for Nanomedicine

The tumor penetration of nanomedicine is a challenge because of NP properties and the tumor microenvironment. Tumor penetration of NPs is dependent on the blood vessel arrangement as well as distribution [14,19,20]. First, the rapid spread of tumor cells results in oxygen and nutrition deficiency, which hasten the development of new vessels and cause abnormal vasculature and heterogeneity of the blood vessels [21]. These rough blood vessels result in heterogeneous and lessened tumor flow of blood and ultimately restrain NPs perfusion at the tumor site [22]. Initially, owing to lessened blood flow, the tumor penetration of nanoparticles becomes hindered [23,24]. The latest study revealed that the nanocarrier’s effect of enhanced permeability and retention (EPR) was related to blood vessel damage and further associated with the tumor density and blood flow [19]. Second, in the middle of tumor tissue, substantial stress is exerted by proliferating cancer cells resulting in the compression of lymphatic and blood vessels, resulting in vessel failure [25] and causing functional lymphatic and blood vessels to be concentrated at the tumor sides [26]. Vessels are distributed heterogeneously from the periphery to the center of the tumor, thus amplifying the poor penetration of NPs.

Extravasation from the vascular system into cancer interstitial gaps, high IFP, and dense ECM restrict nanoparticle delivery [11,12,18,27]. The ECM comprises proteoglycans, glycoproteins, polysaccharides, and proteins produced by endothelial, epithelial, and stromal cells. The ECM has very narrow pores, i.e., less than hundreds of nanometers, which hinders the delivery of nanoparticles by electrostatic interactions and steric restriction [28,29,30]. IFP in healthy tissues is 0 mm Hg, and solid tumors exhibit IFP of about 5–40 mm Hg. However, in some cases, its value reaches 75–130 mm Hg [9,31,32]. The raised IFP lessens the penetration of large-sized nanoparticles and forces them back into circulation [33]. The tumor ECM plays a role in the binding site barrier (BSB) for ligand-modified NPs [34,35]. Initially, the BSB was in antibody transport, captured by cells peripheral to the blood vessels, restraining their tumor penetration [36]. BSB limits the diffusion of NPs, hindering the NPs from reaching deep areas of the tumors due to being captured by the tumor ECM or trapped by stromal cells adjacent to the vessels [37].

Some distinctive features of nanoparticles complicate their transport to tumors. One of the notable features is the nanoparticle size. However, the tumor’s blood vessels are leakier relative to the healthy ones. However, the vessel pores are permeable only at some locations [38], resulting in a difference in the extravasation and distribution of nanoparticles [19]. Therefore, the erupted tumor sites are repaired by NPs perfusing from other areas, enhancing distance and delivery complications. Moreover, alteration in nanoparticle functional groups prompts the capturing of nanoparticles by ECM, thus hindering their delivery to interstitial tumor spaces. Concisely, the surface interactions of the nanoparticles with the surroundings and the narrow pores in ECM restrict their delivery from well-perfused to poorly-perfused areas [39].

The aspects influencing poor penetration include the characteristics of nanoparticles and the biological barriers of tumors [40]. Therefore, efforts are made to rationalize the features of nanoparticles and regulate the tumor microenvironment to lessen the physical barriers, leading to improved tumor penetration.

## 3. Conventional Approaches in Modifying the Tumor Penetration

The conventional strategies to improve the delivery of nanoparticles are classified into (a) tumor environment modulations, (b) extra-cellular matrix tuning, and (c) optimizing the nanomedicine’s physical characteristics. However, these approaches alleviate the poor NP penetration at the tumor site, but their usage is restricted by the interrelated and complex tumor microenvironment and transport cascades.

### 3.1. Tumor Environment Modulations

#### 3.1.1. Vascular Disturbance

The vascular penetrability is increased in order to improve the nanocarrier extravasation in vascular interruption. The blood vessels are disturbed easily by employing physical forces such as ultrasound, radiation, hyperthermia, or any physiological agents, such as vascular disrupting agents. Hyperthermia involves increasing body temperature up to 40–43 °C and is usually applied along with chemotherapy and radiotherapy [41,42,43,44,45,46]. Studies presented that hyperthermia increased the extravasation and perfusion of NPs owing to damaged vasculature and enhanced blood flow [47]. In previous studies, the tumor site is heated directly using heating coils, water baths, and microwaves of low specificity [48]. Nanotechnology combines therapy and heating functions in a single platform. Nanoparticle-based photothermal therapy (PTT) is one of the best methods. In the previous studies, several polymers have been used for packaging or conjugating near-infrared (NIR) investigations or coating photothermal in-organic NPs [49,50,51,52], permitting nanoparticles to penetrate deep in sites away from vasculature exposed to the NIR laser. However, the temperature used in PTT is above 43 °C, which leads to damage and collapse of the vasculature. This impairment obstructs blood flow, thus inhibiting the delivery of other NPs to that region [53].

Local radiation is also considered effective in improving the penetration of nanoparticles. Previous clinical data presented that, in patients with tumors, high dose radiation co-administration resulted in intratumoral accumulation of liposomal doxorubicin (DOX) [54]. Vascular permeability and enhanced blood flow are the two ways to increase transport. Fractionated radiation and extensive single dose radiations induce endothelial cell apoptosis, resulting in vascular collapse and ultimately improving the permeability of NPs [55,56,57]. However, the vascular disruption increases the hypoxia of tumor microenvironments, influencing drug tolerance and tumor recurrence [58].

Ultrasound is usually applied in cancer therapy as a diagnostic method [59]. Several exposure circumstances (duration, pressure, frequency) result in acoustic, cavitational, and thermal effects, improving the penetration of nanoparticles [60,61,62]. One strategy to enhance nanoparticle penetration is the co-administration of nanoparticles and nano/microbubbles, succeeded by ultrasound [63,64,65,66]. Nevertheless, this strategy is ineffective in-vivo due to the short lifetimes of micro/nanobubbles and the need for NPs to co-localize with the cavitation nuclei. Therefore, several “all-in-one” acoustically active transport vectors are used to solve this issue. For instance, one optimal designed structure is the “nanoparticle-microbubble pendant”, with imaging or therapeutic agents filled into nano vehicles, such as polyplexes and liposomes. The NPs can then conjugate to the microbubbles’ surface via coupling reactions or utilizing the biotin-avidin affinity [67,68,69,70,71].

Ultrasound collapses the microbubbles, liberating the pendant liposomes. In addition, the enhanced capillary permeability permits deeper NP penetration at tumor sites. Another approach is to enclose imaging or therapeutic agents in the micro/nanobubbles composed of polymers or lipids [72,73,74]. Unlike lipid-based micro/nanobubbles, polymer-based nano/microbubbles enclose hydrophilic and hydrophobic substances and comprise higher stability and loading space [75,76,77,78]. Early studies presented that ultrasound damaged smaller vessels at the tumor site, blocking recurrent administration of the drug [79].

VDAs result in the selective and speedy shutdown of established tumor vasculature, resulting in successive tumor cell death [80]. 5,6-Dimethylxanthenone-4-acetic acid enhances tumor vessels’ permeability by inducing tumor necrosis factor-promoting NP penetration [81]. Combretastatin A-4 phosphate increases accumulation and rationalizes the distribution of inorganic NPs and liposomes [82,83]. Moreover, a decrease in tumor-associated platelets by antiplatelet antibody also results in the eruption of vessels. Platelets inhibit bleeding and hold the tumor vasculature integrity [84]. The depletion of systemic platelet, up to a large extent, dramatically enhances anticancer drug penetration [85]. Tumor-targeted transport of an antiplatelet antibody, i.e., R300, increased nanoparticle penetration and improved vascular breaches [86]. However, similar to physical forces, the administration of VDA hinders blood supply and causes tumor vessels to collapse.

As external modulators, physiological or physical forces temporarily enhance capillary permeability and reduce ECM density, improving nanoparticle penetration. However, the damaged vessel hinders the blood supply and may cause vessels to collapse, significantly impairing further treatment.

#### 3.1.2. Normalizing the Circulatory System

The drug delivery is improved by normalizing the vascular networks and restoring the blood supply, causing enhanced penetration and optimized distribution of drugs at the tumor site [87]. In the case of tumors, an imbalance of antiangiogenic and proangiogenic factors results in dysfunctional vessels [88,89]. Proangiogenic factors are overexpressed in cancer, leading to abnormal vascular networks. Antiangiogenic and proangiogenic elements are balanced to normalize the vascular network at the tumor site [90].

Antiangiogenic and anti-vascular endothelial growth factor agents are the chief strategies in clinical and research settings [91]. Treatment with antiangiogenic factors results in notable alterations in the structure and functions of blood vessels, involving decreased IFP, enhanced vessel pericyte coverage, and hindrance of immature vessels [26,91]. Research has presented that vascular normalization modifies the penetration and distribution of macromolecules as well as small substances [87,92,93]. Thus, vascular normalization is considered the best substitute for EPR failure in the human body. Though vascular normalization improves the penetration of macromolecules and small agents, the modifications of NP penetration depend on the vascular densities at the tumor site and nanoparticle size. Normalization of blood vessels may compromise the transvascular delivery of more significant NPs due to the lessened pore size of the vascular walls [14,94].

Normalized vessels only allow the transvascular transport of nanoparticles smaller than 12 nm [95]. Some studies proposed that nanoparticles as large as 40 nm can be transported; however, this size limitation is exceeded by synthetic nanoparticles [96,97]. The initial tumor vascular density also affects the results of chemotherapy [98]. Relative to a low vascular density tumor, a high vascular density tumor and recruited pericytes express a better response to bevacizumab (BVB)-accompanied chemotherapy. Consequently, tumor manipulation for the increased antineoplastic nanotherapeutic transport needs more research.

### 3.2. Extra-Cellular Matrix Tuning

**Figure 2:**
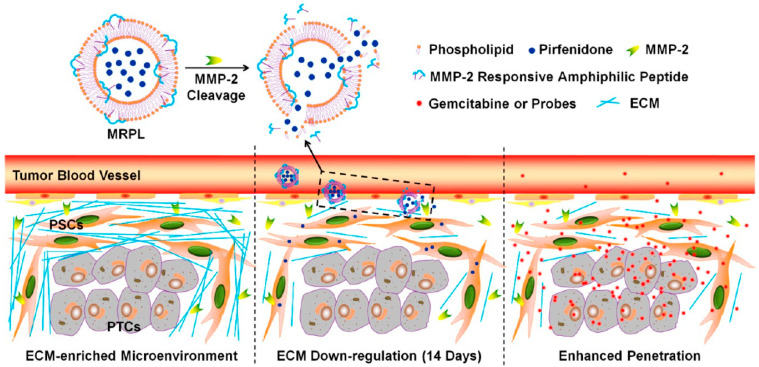
Suggested action mechanism of MMP-2 Responsive Peptide Hybrid Liposome (MRPL) for down-regulation of ECM in pancreatic tumors. Re-printed with permission from reference [99]. Copyright (2017), American Chemical Society.

The extra-cellular matrix (ECM) is one of the main obstacles hindering deep nanoparticle penetration. ECM is adjusted by suppressing its synthesis and deteriorating the existing ECM (Figure 2) [99,100]. Tumor stromal cells are regarded as alternative targets for ECM modulation as they are primary producers of ECM [101,102].

ECM formation can be stopped by using molecular targeting drugs that hinder transforming growth factor- (TGF-), hedgehog signaling, and platelet-derived growth factor- (PDGF-) receptors [103,104,105,106]. PDGF-receptor inhibitors decrease IFP by hindering stromal cells as well as pericytes, thus enhancing the nanoparticle transcapillary delivery in the tumors [103]. TGF-inhibitors are administered in combination with nanoparticles, as they hinder the pericyte differentiation treatment of endothelium even at tiny concentrations [107,108,109]. Furthermore, TGF-inhibitors can manage ECM by reducing collagen I (Col I) content, ultimately improving nanoparticle penetration [110]. In the case of poorly perfused pancreatic tumors, the hindrance of hedgehog signaling disturbs the vascular density and desmoplastic stroma, facilitating the transport of small chemotherapy drugs [106]. However, it is worth considering that the effect of these inhibitors depends upon the tumor type. In the CT26 model, inhibitors have a moderate impact, whereas in the BXPC3 model, the tumor penetration is greatly improved due to the varying vasculature phenotype of these models [111]. Hedgehog signaling is exhibited in around 30% of human cancers such as medulloblastoma, cervical cancer, ovarian cancer, prostate cancer, lung cancer, pancreatic cancer, melanoma and basal cell carcinoma [112]. Thus, the hindrance of signaling pathways is effective merely in specific cancers.

Another approach for the regulation of tumor ECM is the reduction in existing stromal barriers [11]. ECM comprises several stromal barriers such as Col I and hyaluronic acid (HA). The breakdown of ECM lessens frictional resistance as well as IFP, thus improving nanoparticle delivery. A major ECM component has been highly expressed in several tumors [113,114]. Hyaluronidase has been utilized for the breakdown of HA. Intratumoral injection of hyaluronidase lessens IFP, facilitating the penetration of nanoparticles, small-molecule drugs, and antibodies [115,116]. In addition to HA, ECM contains Col I, which creates a dense network by connecting tumor cells to the cell–matrix via cell–cell interactions [117,118,119]. Degradation of Col I weaken the collagen network, facilitating nanoparticle penetration. Pretreatment with collagenase or losartan, i.e., angiotensin-II receptor antagonist, mimics the penetration of polymer-based nanospheres, liposomes, and macromolecules [120,121,122]. The depletion of collagen was significantly associated with the tumor cell’s invasion and metastasis [123,124,125]. Tiny fragments of HA deteriorated by hyaluronidase also stimulate angiogenesis [126,127]. Thus, the degradation of ECM should be regulated to hold the balance of tumor progression. 

Another approach for regulating ECM is the degradation or re-education of stromal cells [128]. Gold nanoparticles (Au-NPs) are said to be a template to deliver RNA or biological drugs effectively to regulate the pancreatic stellate cells without producing cancer metastasis [129]. Furthermore, gold nanoparticles transform PSCs and quiescence of activated cancer-associated fibroblasts (CAFs) [130,131,132]. Degradation and re-education of stromal cells such as CAFs and PSCs hindered ECM hyperplasia, stimulating the penetration of drugs and their therapeutic outcomes. Furthermore, this approach has not observed any metastatic stimulation, showing that stromal cell regulation is an ideal strategy for ECM regulation. 

### 3.3. Optimizing the Nanomedicine Physical Characteristics

The physical characteristics of nanoparticles, including shape, size, and charge, affect their in-vivo behavior, so efforts are made to evaluate the ideal characteristics of nanoparticles to improve their tumor penetration. NPs having optimized characteristics express improved penetration, yet it is confined by some aspects of drug delivery such as accumulation and circulation.

#### 3.3.1. Nanocarrier Size

The size of nanoparticles is considered a significant aspect in evaluating their penetration because resistance in flow is due to the thin gaps in the vessels, ECM, and the density of cancerous cells [38]. After extravasation in the tumor tissue, the penetration depth has been chiefly facilitated by the diffusion balance [133]. Still, the in-vivo penetration of nanoparticles is challenging as the size of NPs influences other aspects such as accumulation and circulation [134,135].

The NP delivery across biological barriers is regulated by various methods depending upon the size of nanoparticles. For example, lymphatic uptake is used to deliver NPs smaller than 5000 nm, endocytosis uptake delivers NPs smaller than 500 nm, and paracellular passage transport NPs smaller than 50 nm [136]. Smaller NPs, due to their low diffusion scales, penetrate deeper relative to larger nanoparticles; however, their penetration is restricted by high elimination rates owing to less retention at the tumor site [133]. Consequently, the balance between clearance and penetration shows how deep the nanoparticles can be delivered. Blood circulation and tumor accumulation in vivo studies are also measured to evaluate the overall transport effect. The size of nanoparticles affects the circulation time to some degree. As compared to smaller nanoparticles, larger ones (greater than 200 nm) show fast clearance by the complement system and subsequent reticuloendothelial system [137,138]. Small NPs (smaller than 5 nm) are quickly cleared by glomerular filtration and renal excretion [139,140,141]. Passive accumulation through the EPR effect is also associated with the size of NPs [142,143]. 

The EPR effect variation can be due to varying methods adopted to cross the vessels using varying-sized nanoparticles. Larger nanoparticles leave the blood circulation during the venting of vessels; after this eruption, the vents close, ultimately hindering diffusion back into the blood circulation [19]. On the other hand, the smaller nanoparticles swiftly diffuse from vessels to the perivascular sites and vice versa. Blood circulation and tumor accumulation are the prerequisites for tumor penetration. In the case of in-vivo delivery, the size of the nanoparticle is optimized by combining all the aspects influencing the fate of nanoparticles.

**Figure 3:**
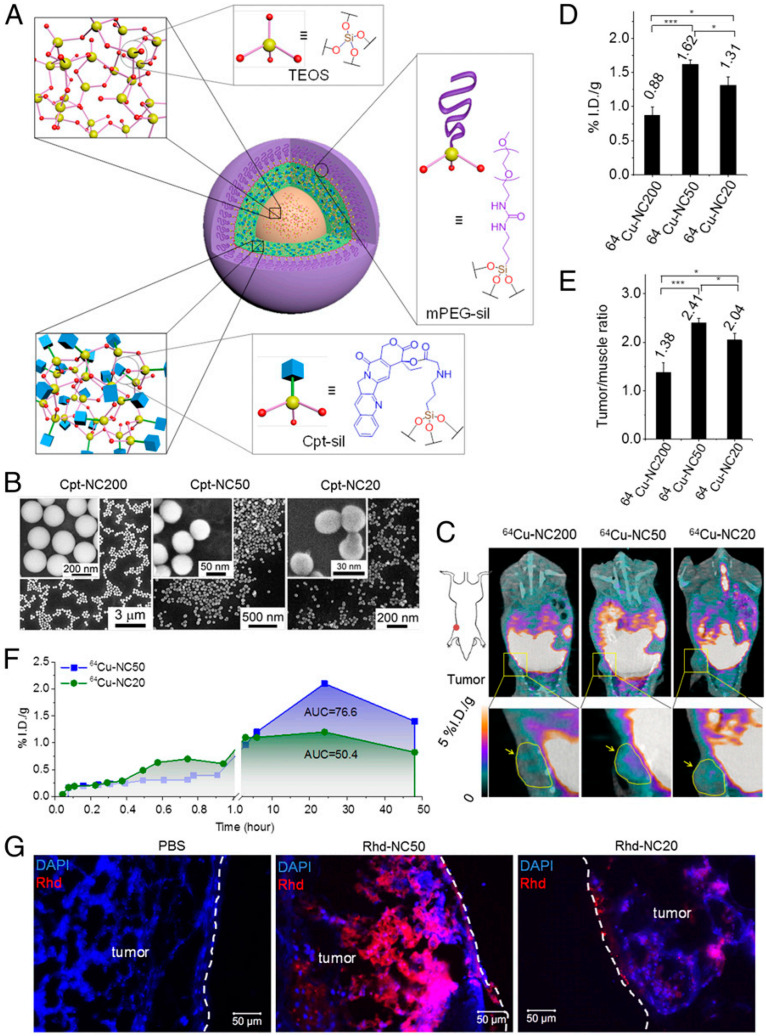
(**A**) Illustration of the size-controlled drug silica nanocarriers. (**B**) Cpt nanocarriers SEM scans. (**C**–**E**) In vivo bio-distribution of ^64^Cu-NCs in mice with MCF-7 human breast tumors. All of the data are represented as the average ± SEM and analyzed by one-way ANOVA (Fisher; 0.01 < * *p* ≤ 0.05; *** *p* ≤ 0.001 (**F**) Kinetic observation of tumor growth of ^64^Cu-NC20 and ^64^Cu-NC50. (**G**) Ex vivo tumor clearance analysis in MCF-7 tumors. Re-printed with permission from reference [133]. Copyright (2014), Proceedings of the National Academy of Sciences (PNAS).

Several in vivo as well as in-vitro studies are made to determine the optimal size range of NPs for penetration [133,134,144,145,146,147,148,149,150]. Different optimal size range is expressed for different nanoparticles, such as 30nm for PEG-b-PLA micelle [144], from 2 to 6 nm for ultrasmall gold nanoparticles [146], 50 nm for drug-silica nanoconjugate (Figure 3) or larger Au NPs [133,151], and about 70 nm for PLGA-NPs [152]. The selection of size is yet to be studied, which may be due to several restrictions of the size optimization approach. (1) The size of nanoparticles is considered one of the major aspects influencing tumor penetration. Varying NPs are composed of distinct substances and have various constituents, so the optimal size range from one research is applicable to only a particular kind of nanoparticle. (2) The animal species and the kind of tumor also influence the degree of tumor penetration. Some large-size NPs express good permeability in loose tumors, whereas the penetration of small-size NPs is good in the case of both flexible and dense tumors [144]. Thus, size optimization of NPs is a good strategy for screening substances, but novel studies must focus on the productive methods to determine the appropriate nanoparticle sizes with modified tumor therapies.

#### 3.3.2. Nanocarrier Shape

Spherical nanoparticles are frequently used in drug transport. The shape of NPs also influences the in vivo fate, such as tumor accumulation, blood circulation, penetration, and cell uptake [153,154,155]. In addition to spherical NPs, other shaped nanoparticles, such as nanocages, nanodisks, nanorods, nanotubes, and worm-like or filamentous micelles, are evaluated to modify NP tumor penetration [156]. NPs of varying shapes have varying penetration capabilities and move across the gaps of dense ECM and tumor cells distinctly, depending upon their particular penetration capability [157]. However, the shape effect also differs in nanoparticles of varying composition and varying tumor models. For instance, the in-vitro analysis of a 3D spheroid model displayed that the nanodisk of height and diameter of 100 and 325 nm, respectively, comprising nano-scale geometry of Jet and Flash Imprint Lithography of PEG hydrogel, have enhanced penetration relative to a nanorod of 400 nm length and 100 nm diameter [147]. Another study presented that a nanorod of diameter and length around 15 and 54 nm, respectively, composed of CdSe/CdS quantum dot (QD), delivered effectively to perivascular areas at deep tumor sites with ortho-topic E0771 mammary cancer as compared to the spherical nanoparticle of diameter about 35 nm [158]. One of the in-vivo studies presented that gold (Au) nanocage and Au nanorod express enhanced penetration in a murine EMT6 breast tumor model compared to Au nanodisk and nanosphere of the same diameter [159].

**Figure 4:**
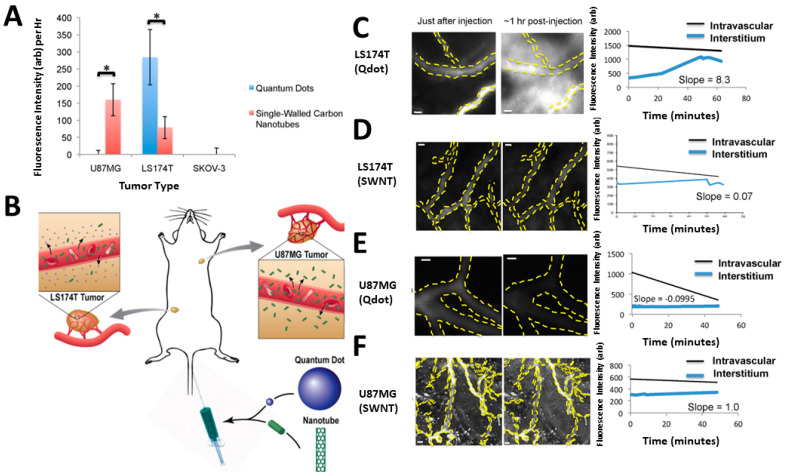
(**A**) The quantum dot extravasation contrasted to SWNTs for every single cancer form. * Indicates *p* < 0.05. (**B**) An overview of the NP outburst. (**C**) At around 1 h of the treatment, the quantum dot in LS174T cancer strongly exits the vascular system. (**D**) Furthermore, SWNTs are not extravasated as compared to the quantum dot. The scale bars of (**C**,**D**) denote 20 μm (**E**) In U87MG cancer, the quantum dot does not extravasate out from the circulation. Scale bar signifies 10 μm (**F**) Autofluorescence is seen in the SWNT scenario in the photos taken soon following administration. Scale bar signifies 50 μm. Re-printed with permission from reference [161]. Copyright (2012), American Chemical Society.

Furthermore, the delivery of nanorods is associated with their aspect ratios (ARs). According to an observation, nanorods with an AR of 3.5 express enhanced penetration capability relative to AR of 7 or 16.5 [160]. However, these results may differ in varying tumor types. A single-walled carbon nanotube (SWCNT) of diameter and length around 2–3 and 200 nm, respectively, express better penetration in U87MG tumor relative to the spherical QD of diameter around 20 nm, whereas the results are the opposite in LS174 T tumor (Figure 4) [161].

Distinct cross-linked and/or inorganic hydrogels, which create rigid frameworks, self-assemble NPs from flexible polymer to form variable and dynamic frameworks such as worm-like or filamentous micelles [162,163,164]. In the case of tumor inhibition, filomicelles express enhanced effectiveness as compared to spherical NPs, probably due to better tumor penetration and prolonged circulation [163,165,166,167,168,169]. Lengthy filamentous micelles are stretched by the vascular system in these conditions, leading to fewer cells stuck throughout the bloodstream and increased possibilities of transportation via spaces among endothelium [153,164].

It is stated that non-spherical NPs express enhanced permeability relative to their spherical counterparts. Some benefits of NPs include: (1) the shape of NPs affects its extravasation, perhaps because of varying movement kinetics among spherical and non-spherical NPs [170]. The dynamics of non-spherical NPs are complex compared to spherical NPs. This is because of a notable marginal number relative to spherical NPs, adding to the number of these NPs interacting with the vessel walls and increasing the chance of extravasation [171]. (2) The aspect ratio of non-spherical NPs is regulated to modify their delivery through porous media and pores within the tumor ECM and endothelial cells [160]. 

The conclusion of the studies mentioned above depends upon the kind of tumor and nanoparticle matrices. Moreover, the shape of NPs influences cell adhesion, nanoparticle distribution in the tumors, blood circulation, cell uptake of nanoparticles, and tumor accumulation [172,173,174,175]. For instance, gold nanorods express more effective uptake by macrophages than Au nanospheres, resulting in quick nanoparticle clearance after injection [176]. Thus, optimizing the shape of NPs depends on the various biological cascades that occur during transport.

The majority of invading immune cells are macrophages, which are typically called tumor-associated macrophages (TAMs). TAMs commonly express high levels of macrophage mannose receptors (MMRs), which NMs might benefit from in terms of improving macrophage identification and incorporation. Additionally, such receptors are present in normal macrophages and monocytes, playing an important part in the immune system processes. CD206 is a receptor highly expressed on TAMs and is conveyed in the M2 phenotype compared to the M1 phenotype in humans [174].

#### 3.3.3. Nanocarrier Surface Characteristics

As stated above [177], surface properties, including hydrophobicity, ligand modification, and charge, affect the biological features of NPs. The surface properties of NPs dictate the interactions between NPs and the tumor ECM or blood vessels, ultimately influencing their delivery to the tumor. In addition, the surface properties affect the in vivo outcome of NPs. The hydrophobicity and the hydrophilicity of NPs can be modified by the loading density of PEG or PEG substitutes on the NP surface. The hydrophilicity of the nanoparticle surface upregulates with the increased loading density of PEG. The thickness is regulated by modifying the PEG content and PEG length. An increase in density improves the NPs tumor penetration, which is validated because the PEG-b-PLA NP had about double the PLA or PLGA penetration distance [178]. 

It is worthy to note that polystyrene NPs conjugated with PEG express better permeability through the blood–brain barrier relative to polystyrene NPs with a low quantity of PEG [179]. The increase in permeability is because of sufficient PEG shielding, which hinders adhesion between extra-cellular spaces and NPs and decelerates cell uptake by cancerous cells, permitting the NPs to perforate more deeply. However, high PEG content can affect the function of the ligand [180]. For example, a PEG loading of around 50% reduced the targeting capability of RGD-modified liposome relative to the liposome with 10% PEG density [181]. Furthermore, high PEG density significantly inhibits cellular uptake of NPs by cancerous cells, thus lessening their permeability [182].

Another aspect that regulates the interaction between tumor ECM and NPs is the surface charge. The impact of surface charge on penetration may differ in varying models, and it is sometimes contradicting. Relative to anionic or neutral NPs, cationic NPs target tumor endothelial cells and show enhanced vascular penetration [183,184,185,186]. Still, cationic NPs stick to the tumor cells, reducing their diffusivity [29]. Thus, the anionic or neutral NPs are considered best for tumor permeability. However, several investigational outcomes contradict the conclusion mentioned above. A comparative study of PEG-b-PLA NPs coated with distinctly charged lipids showed that cationic NPs exhibit effective permeability in both the in vivo tumor model and in vitro 3D tumor spheroid model compared to anionic and neutral NPs [187].

Three-dimensional cancer models may accurately mimic certain critical aspects of real cancers, making them a valuable resource for (i) quite accurate preliminary NMs testing and (ii) identifying options with the best prospects of achievement, which will then be tested in vivo. Because of its predictive capability, 3D models could reduce the number of animals used in preclinical investigations, permitting the 3Rs to be followed. The pros of 3D in vitro cancer models include: (i) replicable 3D design, (ii) increased cell–cell and cell–matrix interactions, (iii) various cellular relocations in space, and (iv) effective for drug resistance mechanisms. Furthermore, the shortcomings are (i) more costly than in vitro 2D models, and (ii) more complicated culture models. On the other hand, the pros of 3D in vivo cancer simulations include: (i) greater reproducibility of physiological conditions and eminent biology, (ii) easy growth of cancer, (iii) study of tumor development, and (iv) reproducibility. Additionally, the cons are (i) high costs, (ii) concerns about animal welfare principles, and (iii) not all human targets have an animal homolog target [147,188].

One of the studies presented that in a 3D tumor spheroid model combining murine breast tumor 4T1 cells with stromal 3T3 cells, silica nanoparticles with higher anionic charge (−40 mV) expressed more profound permeability compared to smaller anionic charge nanoparticles (−20 mV) [188]. Analogous trends had been noted by other groups [189,190]. An unexpected result can be due to the balance between transcellular transport and cell adhesion. Though the NPs with positive charge pass through the transcellular pathway, these NPs also adhere to the tumor cells [191]. However, charged NPs are not the best choice for in vivo applications owing to their shorter circulation time and greater liver clearance relative to the neutral ones [192,193,194].

Precisely, nanoparticle features are associated with their biological functions, yet it is challenging to select a widely acknowledged charge, size, and shape. First, this difficulty is because the specific features of NPs and the unique tumor microenvironments of varying tumors lead to conflict in conclusions regarding rationale features. Secondly, the fate of NPs after administration is complex, and rationale features for varying delivery cascades may conflict. Thus, the optimized characteristics for permeability in one transport method may not apply to another. In summary, optimization of components only partly resolves the issue of poor NP penetration.

## 4. Approaches to Improve the Tumor Penetration of Nanomedicines

There are different strategies for improving the nanomedicine penetration of tumors, such as (a) advanced approaches, and (b) nanomedicine flexibility integrating several tasks.

### 4.1. Advanced Approaches

Due to the complexity of transport cascades, approaches such as improving nanoparticle characteristics and modifying tumor microenvironments have several pros and cons. The modification of a tumor microenvironment either by erupting tumor vessels or the adjacent microenvironments improves the tumor perforation of nanoparticles but has limited successive therapeutic administrations. Furthermore, this particular strategy demands physiological agents or external physical forces, thus elevating the financial load on patients. On the other hand, improving the characteristics of NPs includes compromising on various aspects, as the optimized features affect the transport cascades in conflicting means. For instance, the small-sized NPs display good penetration, but they cannot be used in-vivo due to their quick renal clearance. To cope with these issues, novel approaches have been developed by utilizing tailored nanoparticles.

Cancer stem cells are a type of cancerous cell that can initiate new tumors and induce recurrence. These cells are derived from differentiated cells or localized progenitors of adult tissue somewhere at the start of the tumor [107]. There are high levels of cancer-associated fibroblasts in stromal cells, and they have a substantial impact on tumor progression via tumor microenvironment regulation [101]. Bilateral intercellular interaction between cancer-associated fibroblasts and cancer stem cells is critical to keeping the desired tumor microenvironment cell population balance. CAFs, on the other hand, are the principal cause of multipurpose interleukins (IL-6, IL-8) that modulate cancer stem cell–differentiated cancer cell balance and tumor microenvironment neo-angiogenesis. Furthermore, extra-cellular vesicle signal transmission can also drive paracrine cancer-associated fibroblasts and/or tumor signal transduction [107].

#### Improvement of Trans-Cellular Transport

The experiments to enhance tumor penetration are based on increasing paracellular transport to overcome solid tumor ECM barriers. Unfortunately, though, the outcomes of these techniques depend upon the kind of tumor and NP. Nevertheless, transcellular transport is considered the universal strategy for improving tumor penetration.

The peptides were screened randomly for attachment to prostatic cancer cells using bacteriophage presentation to find polypeptide possibilities containing C-terminal arginine, or rarely lysine, residues in a complementary sequence of R/KXXR/K. [195,196,197]. Prior studies were based on a cell-penetrating peptide (CPP) along with its capability to improve membrane permeability. These peptides also obey CendR [198,199]. As a result, these peptides exhibit less tumor selectivity and can invade healthy cells too. Internalizing RGD (iRGD, CRGDKGPDC), the very first tumor-specific penetrating peptide (TPP) is synthesized by combining the cancer peptide RGD and CendR [200]. iRGD uses a multi-stage strategy to integrate cancer localization and infiltration. Primarily, by interacting with integrin upregulated in cancer cells and angiogenic endothelium cells, the RGD pattern detects the cancer spot [201]. The CendR interacting site for NRP-1 is visible after the proteolysis. This motif aided cancer infiltration and systemic outflow by intercellular administration [202], but in comparison with an RGD-modified PEGylated polyamidoamine (PAMAM) dendrimer coupled with DOX through an acid-sensitive link, it demonstrated improved infiltration and deposition in brain cancers [203,204]. The penetration of some TPPs is revealed such as F3 (KDEPQRRSARLSAKPAPPKPEPKPKKAPAKK), TT1 (CKRGARSTC), Lyp-1 (CGNKRTRGC), iNGR (CRNGRGPDC), CRGRRST and tLyp-1 (CGNKRTR), etc. [205,206,207,208,209].

TPPs have been regarded as a worldwide approach for improving penetration at tumor sites. The characteristics of NPs do not confine the conjugation or co-administration of TPPs. Both inorganic NPs such as silica nanoparticles, bismuth sulfide nanoparticles, iron oxide nanoparticles, QDs, and organic NPs, such as dendrimers, nanogels, protein-based NPs, micelles, and liposomes, have been employed effectively [204,210,211,212,213,214,215]. The reason for the successful use of various nanoparticles, following alteration through TPP can be that transcellular delivery is chiefly peptide-dependent rather than NP. TPP-mediated penetration is noted in several tumor types such as glioblastoma, ovarian cancer, melanoma, breast cancer, prostate cancer, liver cancer, and pancreatic cancer [210,216,217,218,219,220]. It is worth considering that the TPP modification or co-administration improved the permeability of NPs in metastatic brain and lung tumors [221,222,223]. These results showed that TPPs affected several kinds of tumors, a finding consistent with the broad expression of integrin and NRP-1/2 in endothelial cells or/and particular cancerous cells [205,224]. Therefore, TPPs have wide applicability for several NPs and numerous cancer types, proposing the outstanding potential to design a worldwide approach for enhancing NP penetration at tumor sites. 

To promote NP uptake, purposely selected nanostructures containing triggerable TPPs are investigated in addition to simple coupling or TPP co-administration. The enzyme-activated cellular penetration of a matrix metalloproteinase 2 (MMP-2)-responsive nanocapsule was discovered [225,226]. In addition to TPP revision, NPs with transcellular transport ability have been designed. A virion-like NP collected from a dendritic arginine-rich peptide prodrug showed improved penetration and cell uptake [227]. Dendritic peptides stimulated the transduction domain of viral proteins as well as cell membrane disruption. The peptide prodrug has been reformulated in the bloodstream with an acid-sensitive component to protect the arginine group, assuring a long circulation half-life. Acid stimulated the membrane disrupting dendritic peptide capability at the tumor site, which resulted in the dispersal of the prodrug in cancer cells. In vivo research in impermeable SKOV3/R tumors presented that the NPs, employing the transcellular pathway, displayed robust transvascular extravasation and deeply perforated cancerous tissues. NPs such as boronic acid-rich chitosan nanoparticles, cationic nanoparticles, and grapheme nanosheets exhibited worthy transcellular transport [228,229,230]. Therefore, peptide-mediated transcellular delivery is beneficial in various tumors. Furthermore, TPP activatable nanosystems are attained through tailored design for tumor-specific penetration. This approach resolves the issues related to the poor penetration of NPs. 

### 4.2. Nanomedicine Flexibility Integrating Several Tasks

The optimization of nanoparticles involves compromises due to the different and sometimes contrasting demands of NPs in varying transport cascades. The transport cascades following nanomedicine administration are classified as circulation, accumulation, penetration, internalization, and drug release [16]. The best possible characteristics of NPs in every stage are distinct and may oppose each other. For instance, small NPs exhibit better penetration, but they are rapidly cleared and have little tumor accumulation [134]. The NPs PEGylation improves penetration by stimulating the paracellular pathway but inhibits tumor cell internalization [231]. Positively charged NPs show better penetration by enabling secondary transcellular pathways but short circulation time relative to anionic and neutral NPs [192]. Optimization of NPs is confined due to the need for contradictory features of NPs in all the stages of drug transport.

The transformable NPs, which can modify their characteristics according to the demand of transport stages, can be an excellent approach to merging the ideal properties of a transport cascade in a single nanoplatform. The charge and size can be modified easily, so these two characteristics are broadly considered to optimize drug transport. Conventional size-transition designs to improve tumor penetration include size-shrinkable NPs and small and large hybrid NPs, whereas the charge transition is generally from anionic/neutral to cationic. Some nanoplatforms integrate both transitions to obtain better penetration. Additionally, the tumor micro-environment, such as overexpressed enzymes and mild acidity, stimulates transitions. Moreover, endogenous environments and external stimuli such as ultrasound and light can also be employed [232,233,234,235,236,237].

Small and large hybrid NPs are made up of two distinct-sized NPs. The smaller nanoparticle is for penetration, whereas the larger one is for circulation as well as accumulation. The smaller NPs are conjugated with or enclosed in the larger one to synthesize larger hybrid nanoplatforms with lessened clearance onto the liver or renal filtration. Following accumulation at the tumor site via active targeting or the EPR effect, the NPs respond to the stimuli, liberating or separating the smaller NPs from the hybrid. Fukumura and coworkers presented an example of “small-in-large” hybrid NPs [238]. QD with a diameter around 10 nm was enclosed in a 100 nm gelatin nanoparticle, which was disintegrated by the upregulation of MMP-2 in the cancerous area.

**Figure 5:**
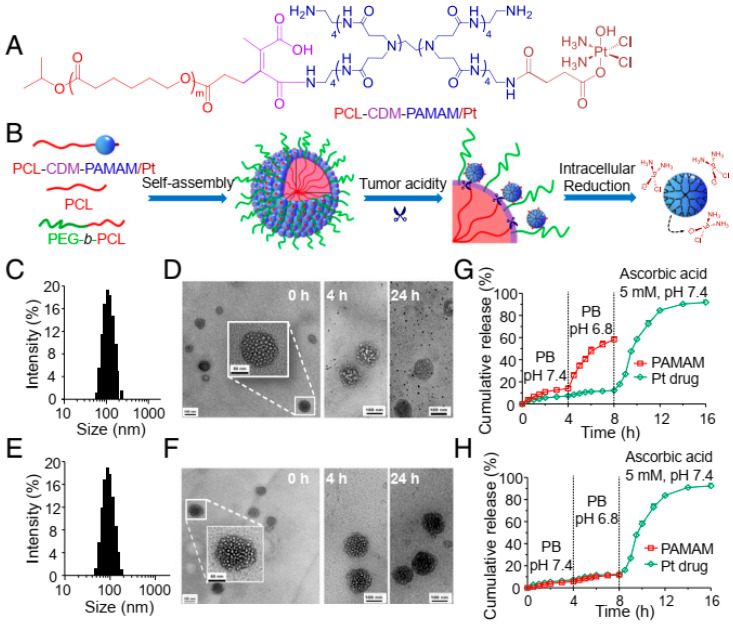
(**A**) The chemistry of PCL-CDM-PAMAM/Pt. (**B**) In accordance with cancer acidosis and the cellular reducing microenvironment, iCluster/Pt self-assembles and alters its structure. (**C**) Size determination of iCluster/Pt and (**E**) Cluster/Pt by dynamic light scattering technique. (**D**) TEM scans of iCluster/Pt and (**F**) Cluster/Pt at pH 6.8 for 0, 4, and 24 h, respectively. (Scale bar, 100 nm and for the Inset images, 50 nm) (**G**) PAMAM and platinum drug release from iCluster and (**H**) Cluster. Re-printed with permission from reference [247]. Copyright (2016), Proceedings of the National Academy of Sciences (PNAS).

Once the gelatin scaffold degenerated, QD was liberated from the hybrid nanoparticles and penetrated tumor tissues in vivo as well as in vitro. Several more “small-in-large” hybrid NPs were presented, such as liposomes, PAMAM dendrimer, polyplex micelles, and dendrigraft poly (L-lysine) (PLL) in polymer micelles, which were activated by numerous stimuli and exhibited better penetration capabilities [239,240,241,242,243,244,245]. Apart from enclosing small NPs in the larger one, smaller NPs self-assemble to create a larger NP [246]. Another design of hybrid NPs includes “small-on-large” hybrid NPs. For instance, platinum prodrug-conjugated PAMAM (PAMAM/Pt) dendrimer conjugate on polymeric nanoparticles employing an acid-cleavable linker (Figure 5) [247]. The hybrid PAMAM/Pt dendrimer presented better circulation features than the free one. Following tumor accumulation, PAMAM/Pt dendrimer was separated from hybrid NPs and presented improved penetration in vivo and in vitro. Gao’s group showed another MMP-2-responsiveness hybrid nanoparticle [248,249,250].

Small DOX-loaded dendri-graft PLL or conjugated Au-NP was conjugated to larger gold-NP (GNP). Then, GNP was degenerated by MMP-2 at a cancerous area, and a smaller nanoparticle was liberated from the hybrid NPs, showing improved penetration. Another form of size-transformable nanosystems includes size-shrinkable NPs. In the case of these NPs, size transition was caused by the shrinkage of nanoparticles. Kohane’s group presented two examples of size-shrinkable NPs [251,252]. Spiropyran-contained monodisperse nanoparticles encountered changeable photo-isomerization following UV radiations at 365 nm that resulted in the shrinkage of nanoparticles from around 150 to 40 nm, thus improving the micelle diffusion. Furthermore, the size shrinkage under irradiation hastened the liberation of a drug from nanoparticles. These NPs exhibited good distribution and regained their dissemination capacity in the same way as UV light stimulation.

Likewise, the charge shift can be used to allow recovered NPs to penetrate the tumor site because of increased transcellular drug transport [187]. For consecutive intra-intercellular NP delivery, a pH-sensitive nanogel with a poly-electrolyte base was produced [253,254]. The active transport facilitated both trans-endothelial and intercellular mechanisms followed by caveolae-mediated transcytosis and endocytosis, resulting in comparatively uniform tumor distribution. Through increased tumor penetration, this prodrug system was considered beneficial in eliminating small solid tumors of around 100 mm^3^ and reverting large tumors of about 500 mm^3^.

The combination of charge and size transition into one nanoplatform could further stimulate permeability using transcellular and paracellular delivery. For the cure of A549 human lung xenograft, Chen and coworkers presented an optimally designed nanoparticle with a charge transition, as well as acid, that activated shrinkage [255]. The combination of positively charged and smaller-sized nanoparticles enhanced the extravasation and penetration of nanoparticles relative to control in vivo. Increased penetration of loading nanoparticles was proved to be beneficial in eliminating A549 tumor xenograft. Furthermore, dual-transformable nanosystems have also been used for siRNA and photosensitizer transport [256]. Mitochondrion-targeting photosensitizer and a predetermined casualty-ligand 1 obstruction were coloaded into an NP made by an acid-breakable PEG shell. 

The detachment of the PEG shell resulted in the charge transition and the size reduction in nanoparticles leading to improved checkpoint inhibition and photodynamic treatment. The resultant systemic anti-tumor immune reaction impeded melanoma progression and minimized the relapse ratio. The customized layout of convertible NPs combined all the desired features in a single platform, offering benefits during various drug delivery cascades and depicting a functional advanced drug transport approach. Nevertheless, the synthesis of existing transformable NPs is uncontrollable as well as complex. Therefore, the clinical applications of these approaches demand further development.

## 5. Conclusions and Perspectives

Numerous eras of nanomedicine advancement have led to several functional nanotherapeutics for diverse needs of drug transport. However, tumor permeability, the most critical step of transport cascades, has been critically inhibited by several biological obstructions such as dense ECM, abnormal tumor vasculature, and IFP. Tumor penetration is also affected by the features of NPs, including charge, size, and shape. 

To cope with these penetration restrictions, numerous approaches were examined, such as optimizing NPs and modulation of the tumor microenvironment. Two direct strategies, including depleting the tumor ECM and disrupting the tumor blood vessels, are employed to improve tumor penetration of NPs. An indirect approach, i.e., normalizing the tumor vessels, improves the penetration of small NPs. In contrast, the modulating effect of the tumor microenvironment depends on the kind of nanoparticle employed and the tumor type. Furthermore, this modulation can disturb the balance of tumor microenvironments resulting in severe difficulties. Optimization of NPs detects the nanoparticle features desired for improved tumor permeability. However, efficient drug transport is extraordinarily complex and demands multiple drug transport cascades, with contradictory requirements concerning optimal features. Nanoparticle optimization requires compromises for effective drug transport. However, it seems impossible to combine all the ideal characteristics in a single platform.

To resolve the issue of conflicting nanoparticle features, two approaches are explored. Targeted transcellular drug transport with a substitutive transport pathway bypasses the biological barriers, and stimuli-responsive nanoplatforms are designed to alter characteristics when exposed to external stimuli or tumor microenvironments. Transcellular drug transport mediated by TPPs exhibits broad applicability by numerous tumor types and NPs and is considered the universal approach for penetration. Rationally designed transformable NPs combine all the features in a single platform, bypassing multiple drug transport barriers. 

Despite having enhanced tumor penetration, clinical applications of rationally designed nanoplatforms demand further improvement. Firstly, an understanding of the tumor microenvironment should be developed, particularly the factors affecting tumor penetration. Moreover, the synthesis of tailored nanocarriers is complex, and their transitions and actions are still ambiguous. The studies should be upgraded to form more effective templates and examine the fate of NPs after in vivo administration. However, efforts are made to increase the nanomedicines penetration, but the penetration depth is uncertain yet, owing to the complexity of biological barriers. Penetration-independent therapeutic techniques can be used as an alternative. For instance, nanoparticle-based immunotherapy, such as immune system activation by co-transport of tumor-specific antigens, immune agonists, and photothermal agents, results in tumor degeneration without tumor penetration [257,258]. Hence, the design of nanoparticles should be able to adjust the demands of distinct therapeutic substances.
